# Studies on seed-borne fungi, *Aspergillus* diversity, and their detection using beta-tubulin gene sequences in *Foeniculum vulgare* seeds in India

**DOI:** 10.3389/fmicb.2026.1782984

**Published:** 2026-06-10

**Authors:** Pranab Kumar Mahata, Regina Sharmila Dass, Lokanadhan Gunti, Pooja Appasaheb Thorat

**Affiliations:** 1 Department of Microbiology, Radha Govind University, Ramgarh, India; 2 Fungal Genetics and Mycotoxicology Laboratory, Department of Microbiology, School of Life Sciences, Pondicherry University, Pondicherry, India

**Keywords:** *Foeniculum* vulgare, seeds-borne fungi, mycology study, *Aspergillus flavus*, mycotoxin exposure, seed health and viability, fungi biodiversity

## Abstract

The presence of fungi in seeds has a negative impact on germination and quality, which eventually leads to fungal deterioration and economic losses. This research inquired into the variety of fungi that were isolated from *Foeniculum vulgare* seeds (70 samples) collected from different regions of India, namely Delhi, Pondicherry, Rajasthan, Uttar Pradesh, and West Bengal. In order to identify the fungal genera, protocols outlined by the International Seed Testing Association (ISTA) (1980) procedures were adopted, and mycological media like, Potato Dextrose Agar (PDA) was used for both qualitative and quantitative analyses. Twenty-five fungal species that belonging to 11 different genera were isolated. *Aspergillus niger*, *A. flavus*, *Mucor* sp., *Mycelia sterilia*, *Penicillium* sp. and Yeast, were the most common types of fungi that were encountered in the present study. This is the first report of five *Aspergillus* spp., being isolated and documented from *F. vulgare*. One of the most significant contaminants, *A. flavus* was frequently isolated, with a toxigenic potential. The other types of fungi that are being reported, include *Fusarium oxysporum*, *F. verticillioides*, *Alternaria alternata*, *Curvularia* sp., and *Trichothecium* sp. Macromorphological and Micromorphological analyses were carried out after incubation at 25 °C for 7 days. Based on the outcome of the present mycological study, *F. vulgare* seeds contain an array of fungal contaminants, which have a potential effect on the health and viability of the seeds. In addition, they pose a serious threat to consumers because of their ability to produce mycotoxins.

## Introduction

*Foeniculum vulgare* is an aromatic and medicinal plant, a member of the Apiaceae family known for its significance in Indian cuisine, commonly referred to as fennel. It is used as a spice and flavoring agent in different parts of the world over, and serves as a traditional remedy for a variety of maladies. The seeds are widely cultivated with India producing 243,666 metric tonnes (Spices Board of India, 2025). India is a significant contributor to the global spice market, as it is one of the top producers of fennel seeds (Meena et al., 2023). For sustainable agriculture, the quality and yield of crops are significantly influenced by seed health. The substantial impact of contaminants, that microbes in general and fungi in particular can have on seed viability and productivity necessitates a comprehensive examination of their presence and effects (Neergaard, 1977). It has been well established through various researches, that fungal contamination in seeds presents a substantial threat to agricultural productivity. Pathogenic fungi produce mycotoxins that can pose significant health hazards to both humans and animals (Bennett and Klich, 2003), in addition to reducing germination rates. The presence of a diverse array of fungal species in asymptomatic seeds is particularly concerning, as it may often go unnoticed, that could potentially disseminate under favorable circumstances. In order to create effective seed treatment and management strategies, it is imperative to understand the influence of fungal diversity on seed health. Seeds serve as reservoirs of a diverse array of microorganisms, including fungi that are both beneficial and harmful. While certain fungi serve as endophytes and enhance the health of plants, others like field, post-harvest, and storage fungi can cause a substantial decrease in the quality of seeds (Rétif et al., 2023). It is imperative to identify and detect fungal species in spice seeds in order to effectively mitigate their adverse effects through the implementation of appropriate and scientific agricultural practices. Standardized methodologies for the detection and quantification of seed-borne fungi have been devised by the International Seed Testing Association (ISTA, 1980; Seed Science and Technology Committee, 2025). By enabling farmers and researchers to conduct reliable assessments of seed health, these protocols facilitate the implementation of preventive measures against fungal infections. The current study has been conducted in a detailed manner to isolate and identify fungal species, most effectively achieved through the use of mycological and fungi-specific media namely Potato Dextrose Agar (PDA) media (Li et al., 2025). Additionally, the study has been undertaken to identify to gain insight related to seed health. The quality of spice seeds, agricultural productivity, and food safety are all at risk due to fungal contamination in seeds. The present study offers valuable insights into the fungal diversity associated with *F. vulgare* seeds, with emphasis to *Aspergilli*, which is the first of its kind, underscoring the significance of effective management strategies and continuous monitoring. Stakeholders in agriculture can ensure healthier seed supplies and enhance overall crop yields by implementing ISTA methodologies and laboratory procedures to assist in the overall seed monitoring. Understanding the effects of fungal contamination can help reduce economic food losses, support the development of disease-resistant plant varieties, and improve post-harvest management practices. The findings of this study will establish a strong foundation for future research focused on preserving the quality and viability of fennel seeds, enabling mycologists, researchers, and policy makers to determine permissible levels of specific mycotoxins in both traditional and commercial farming systems.

## Materials and methods

### Sample collection

**Figure 1:**
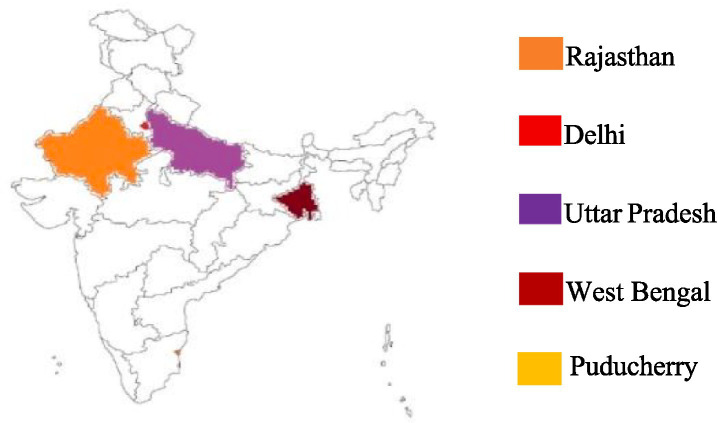
Sample (*F. vulgare*) collection sites from different states of India.

Seventy (70) samples of *F. vulgare* seeds were collected from retail markets in Rajasthan, New Delhi, Uttar Pradesh, West Bengal, and Puducherry, encompassing urban, semi-urban, and rural areas of India (Figure 1). Each sample comprised of 100 g, which were placed in sterile plastic zip-lock pouches, coded. The samples were brought to the lab and preserved at 4 °C before being tested, ensuring careful handling and storage. Samples were subjected to analyses by following standard operating protocols of ISTA (1980) and Fungal Genetics and Mycotoxicology Laboratory.

### Culture-based macro-morphological analyses

Mycoflora analysis of Indian *F. vulgare* seeds were performed agar plating methods recommended by ISTA (1980). Each spice sample was washed three times with distilled water to get rid of any surface contaminants and then surface-sterilized with 1% sodium hypochlorite (NaOCl) for 1 min. Then, sterile distilled water was used to rinse the seeds to wash away traces of any NaOCl. The spice seeds were placed on sterile tissue paper in Petri plates allowed to sit for 5 to 10 min to dry out. *F. vulgare* samples were plated on potato dextrose agar (PDA) media (HiMedia®, Mumbai, India) to isolate the fungi (Shango et al., 2024). The media were also supplemented with 20 mg/1,000 mL of thermostable antimicrobial Chloramphenicol (Sigma-Aldrich, Steinheim, Germany). Twenty-five (25) seeds per petriplate (100 × 15 mm S-line Petriplate Borosil®, Mumbai, India) were placed using aseptic techniques and incubated at 25 °C ± 2 °C for 5–7 days, alternating between 12 h light and 12 h darkness. Spice samples on PDA media generated green, greenish-yellow, ochre/yellow, black, brownish-black, dark-brown, biscuit-brown, and blue fungal colonies after 7 days of incubation. Fungal spore suspensions (20 μL) were produced and placed on fresh PDA media. Full -fledged, seven-day old colonies were viewed using a Stereo Binocular Microscope (Magnus MSZ-Bi, Model: 13 M1009). Microscopical studies, anatomical observation, and identification up to genus level were performed using Fungal Keys and Manuals (Thom and Raper, 1945; Booth, 1977; Klich, 1996; Varga and Samson, 2008) and Monographs (Refai et al., 2014; Glässnerová et al., 2022). Some of the features used in the preliminary identification process involved obverse and reverse colony characteristics, colony size, color and texture, presence or absence of sporulation, and ascomata (in 15–25-day old cultures), etc. The results were expressed as percentage of infection and the frequency of occurrence was calculated (Reddy et al., 2009; dos Santos-Ciscon et al., 2019).

### Quantitative studies of fungal colonization in *F. vulgare* to determine percent incidence and frequency of occurrence

Standard formulae (Incidence (%) = (Number of positive samples / Total specimens analyzed) × 100) to calculate percentage incidence of *Aspergillus* species and other fungi were used, to study quantitative contamination across fennel samples. The percent incidence data helped to determine the infection levels and fungal prevalence in each sample (Shango et al., 2024; Selemani and Madege, 2025).

### Culture-based micro-morphological analyses

Light (Olympus CH20i), Scanning Electron (SE; Hitachi, Model E-1010), and Differential Interference Contrast (DIC; Nikon ECLIPSE Ti-U optical microscope equipped with differential interference contrast optics) microscopy techniques were employed to investigate the isolated fungi. High-resolution photomicrographs were captured using Nikon NIS-Elements Imaging Softwarev4.4 connected with Nikon DS-Fi2 digital camera. The characteristics considered for identification processes were hyphal structure/ nature, presence or absence of septa, conidiophores, shape of the vesicle, number of rows of sterigmata, conidial arrangement, and presence or absence of conidial ornamentation. Microscopic characteristics were analyzed using fungal identification Keys and Manuals (Thom and Raper, 1945; Varga and Samson, 2008). Light microscopic studies were performed at Fungal Genetics and Mycotoxicology Laboratory, Department of Microbiology, scanning electron microscopy at the Central Instrumentation Facility (CIF), while DIC microscopic analyses were performed at Fungal Biotechnology Laboratory, Department of Biotechnology, with all facilities housed within the Pondicherry University.

### Molecular identification of *Aspergilli*

The molecular identification of *Aspergilli* was carried out in addition to morphological studies. Briefly, the steps consisted of fungal DNA extraction, PCR amplification of the gene markers and Sanger’s sequencing of the amplicons. The universal fungal barcode ITS, was used for species-level identification. In addition, functional gene sequences like, β-tubulin gene marker was also used as part of multi-gene phylogeny studies for species-level identification of *Aspergilli*. One of our previous studies (Mahata et al., 2022a) has described the molecular phylogeny of *Aspergilli* and NCBI-GenBank submissions have been completed (Table 1).

**Table 1:** The GenBank Accession numbers obtained from National Centre for Biotechnological Information (NCBI) for the ITS and β-tubulin gene sequences of the *Aspergillus* species isolated and reported from *F. vulgare* from India.

**Isolate code**	**Section**	**Species identified**	**ITS gene (GenBank)**	**β-tubulin gene (GenBank)**
18	*Terrei*	*Aspergillus aureoterreus*	Not available	MN791096
16		*A. terreus*	MN392907	MN791095
61	*Flavi*	*A. flavus*	Not available	MN791106
22		*A. tamarii*	MN326529	MN791098
31	*Fumigati*	*A. fumigatus*	MN264637	MN791103
45	*Nidulantes*	*A. nidulans*	MN309877	MN791101
58		*A. quadrilineatus/ Emericella quadrilineata*	Not available	MN791105
4		*A. latus*	Not available	MN791110
S24	*Nigri*	*A. awamori*	Not available	MN791114
83	*Versicolores*	*A. sydowii*	MN298848	Not available
29	Unassigned	*Aspergillus* species	MN294688	Not available

## Results

### Morphological characterization of fungal contaminants in *F. vulgare* seeds

The determination of percent (%) incidence and frequency of occurrence (colonization frequency) were obtained, which are established procedures in mycological seed testing protocols. This method resulted in the determination of infection frequency for all the fungal genera, which are crucial in seed health testing procedures, which is more emphatic and widely accepted in taxonomic studies (Selemani and Madege, 2025). In addition, fungal diversity was also obtained by classifying the seed-borne fungi into their representative genera as *A. alternata, Curvularia, Drechslera, Fusarium, Mucor* sp.*, Mycelia sterilia, Neurosprora* sp., *Penicillium* sp.*, Trichothecium* sp., and Yeasts as a preliminary step based on Stereo-binocular and light microscopic studies.

**Figure 2:**
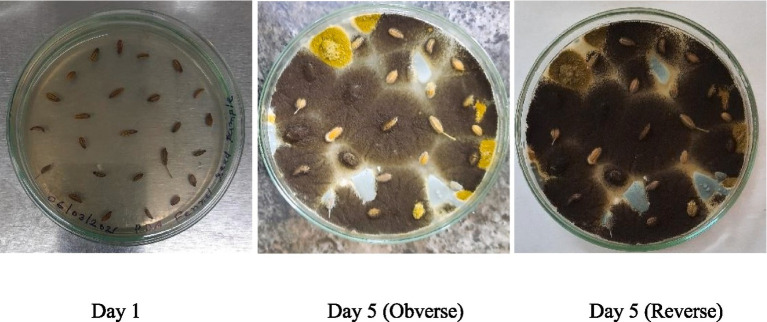
The sample S56-FOEV56 (2nd set study*) sample showed fennel seeds and infected seeds after 5 days of incubation. *Sample of fennel seeds used for second time agar plating/testing.

**Figure 3:**
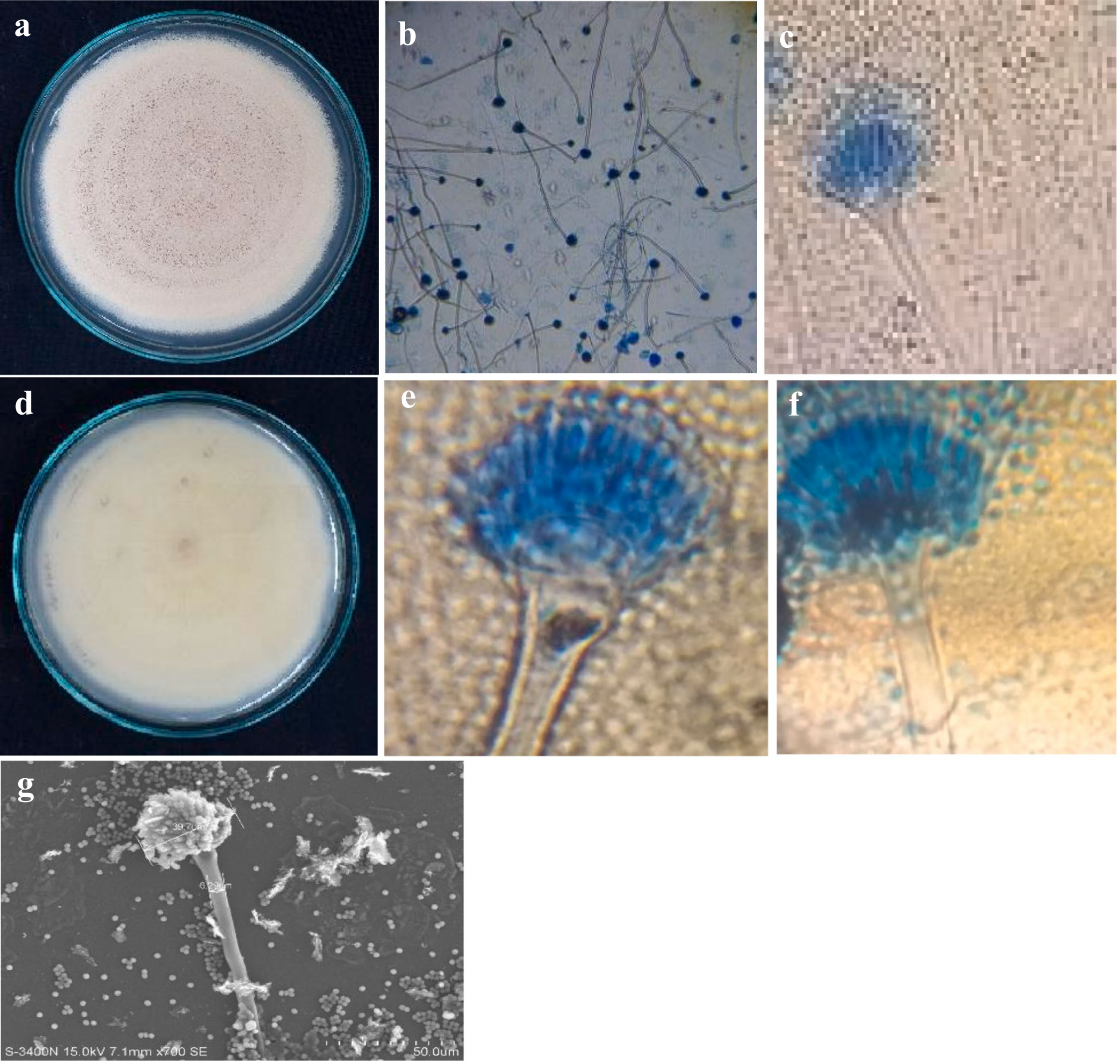
*Aspergillus aureoterreus* (Acc. No. MN791096) colonies, conidiophores, vesicles, phialides, and conidia. **(a,d)** Obverse and reverse views of the fungal isolate, incubated at 25 °C on PDA medium; **(b)** (CLM) Hyphae and elongate conidiophores; **(c,e,f)** (CLM) Smooth, long, colorless conidiophores and columnar, biseriate conidial heads with hemispherical vesicles, and conidia; **(g)** (SEM) Conidiophores. Scale bars: **(g)** = 50 μm. The figures **(b,g)** have been published in Mahata et al. (2022a).

**Figure 4:**
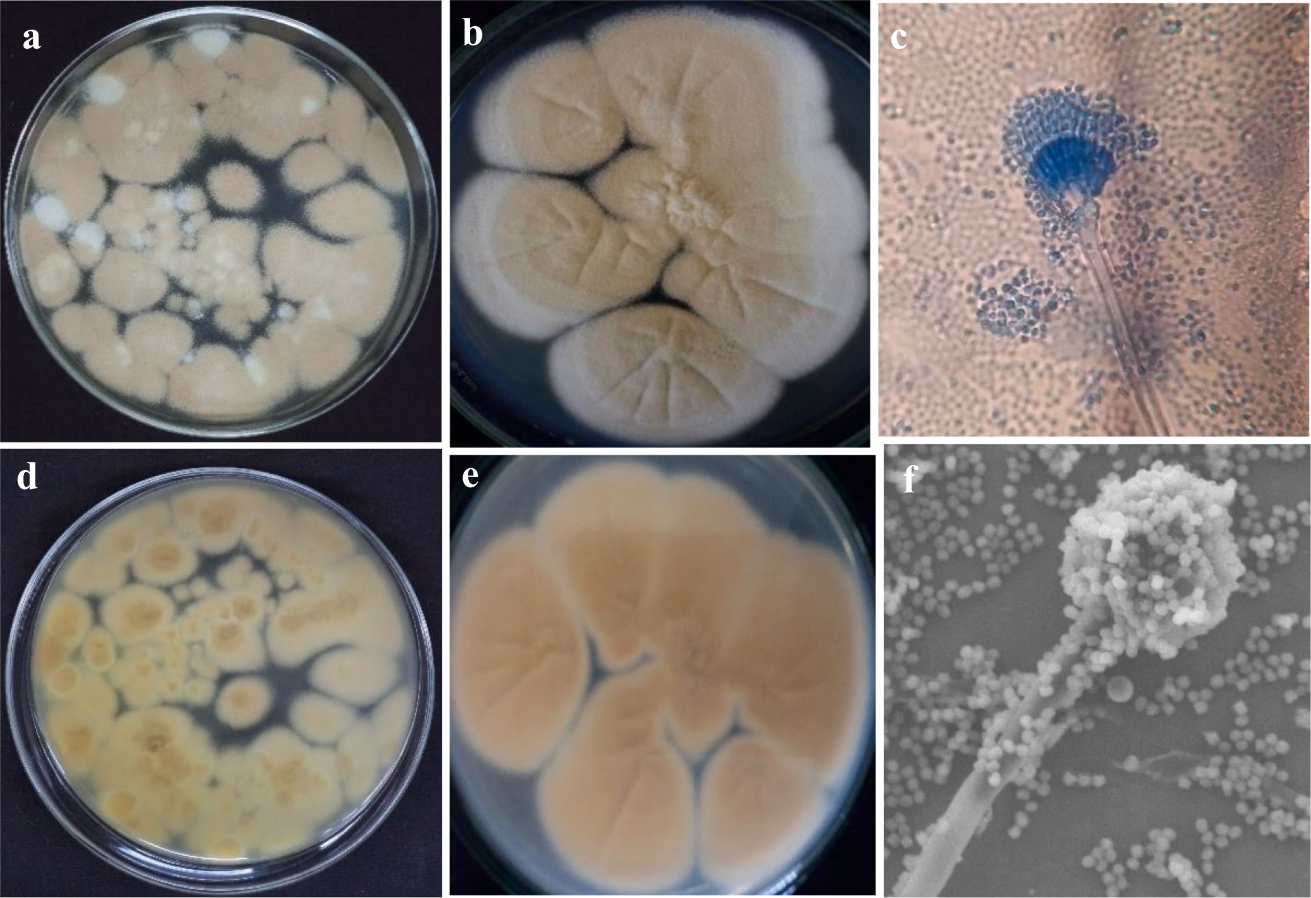
*Aspergillus terreus* (Acc. No. MN791095) colonies, conidiophores, vesicles, sterigmata, and conidia. **(a,b,d,e)** Obverse and reverse views of the fungal isolate, incubated at 25 °C on PDA medium; **(c)** (CLM) Conidiophores enlarged; **(f)** (SEM) Smooth, colorless conidiophores and columnar, biseriate conidial heads with globose vesicles, and small, smooth, globose conidia. Scale bars: **(f)** = 50 μm. The figures **(b,e,f)** have been published in Mahata et al. (2022a).

**Figure 5:**
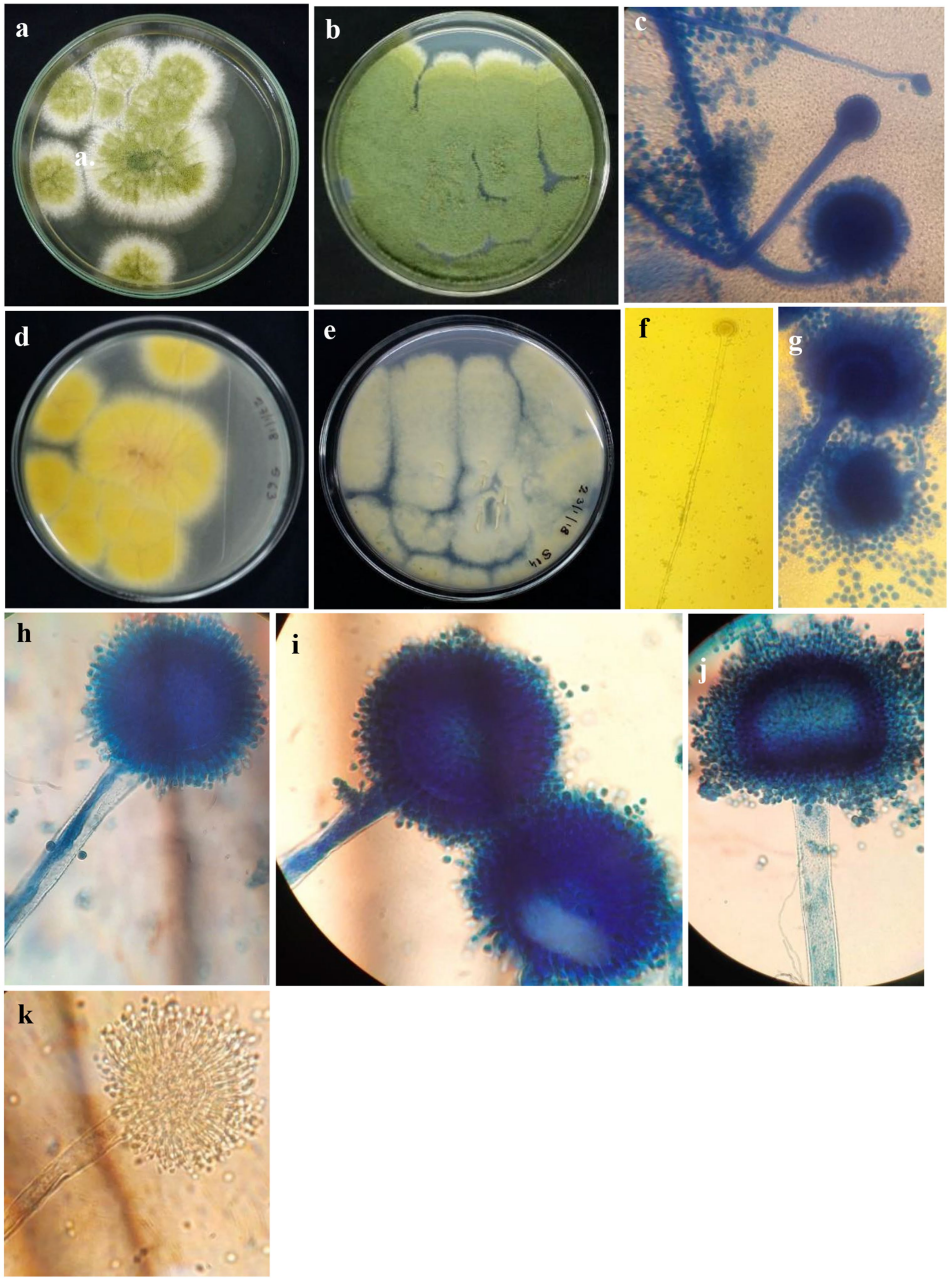
*Aspergillus flavus* (Acc. No. MN791106) colonies, conidiophores, vesicles, and conidia. **(a,b,d,e)** Obverse and reverse views of the fungal isolate, incubated at 25 °C on PDA; **(f,k)** (CLM) Conidiophores, unstained; **(c,g–j)** (CLM) Conidiophores, stained, double-walled conidiophores and with globose or hemispherical vesicles, biseriate sterigmata, and conidia. The figures have been published in Mahata et al. (2022a).

**Figure 6:**
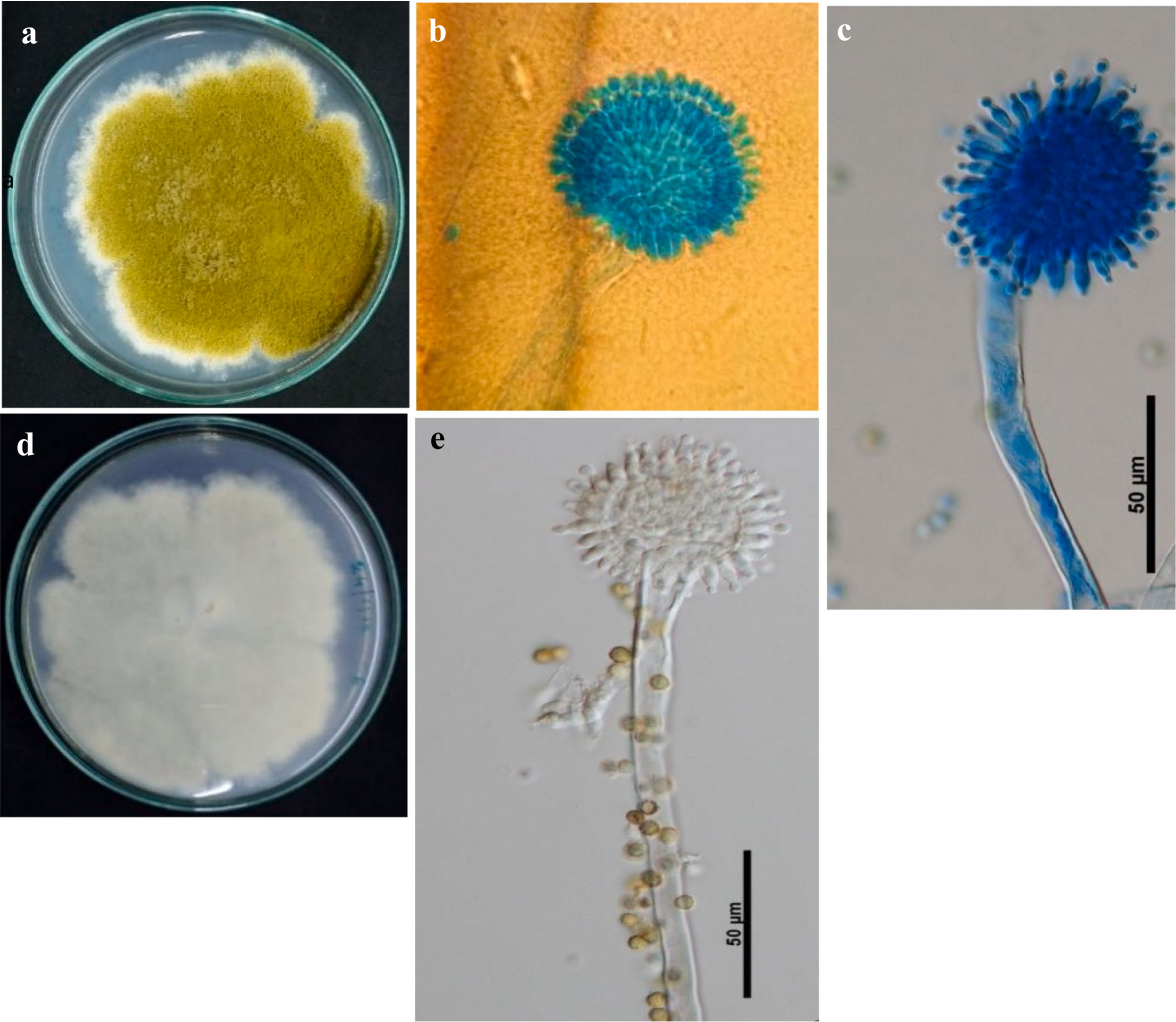
*Aspergillus tamarii* (Acc. No. MN791098) colonies, conidiophores, vesicles, sterigmata, and conidia. **(a,d)** Obverse and reverse views of the fungal isolate, incubated at 25 °C on PDA medium; **(b)** (CLM) conidial head enlarged; **(c,e)** (DIC) conidiophores, globose, radiate conidial heads, globose vesicles with uniseriate sterigmata, and globose, rough conidia. Scale bars: **(c–e)** = 50 μm. The figures **(a–e)** have been published in Mahata et al. (2022a).

**Figure 7:**
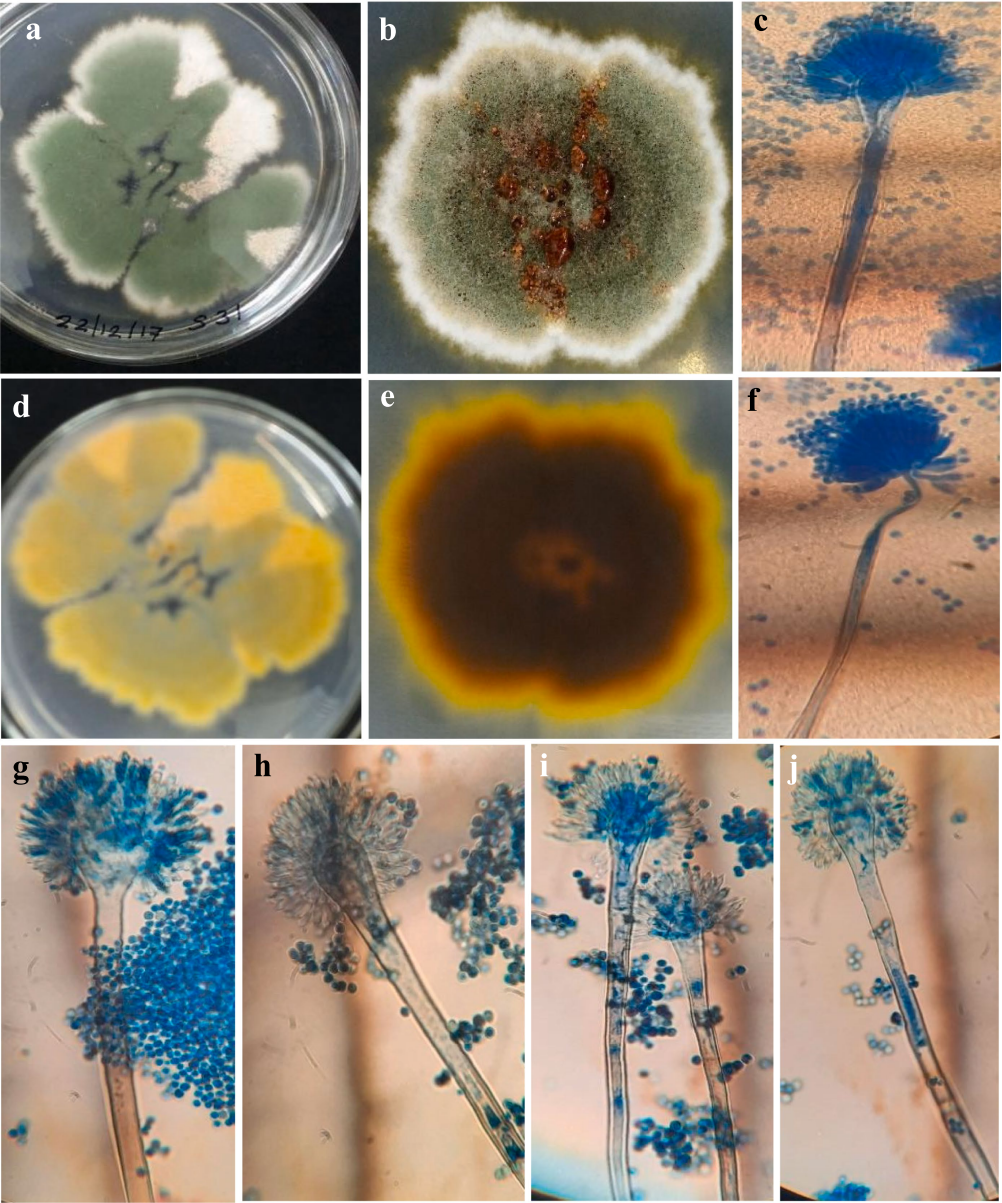
*Aspergillus fumigatus* (Acc. No. MN791103) colonies, conidiophores, vesicles, and conidia. **(a,b,d,e)** Obverse and reverse views of the fungal isolate, incubated at 25 °C on PDA; **(c,f–j)** (CLM) conidiophores with bottle-shaped vesicles, uniseriate sterigmata, and conidia. The figures **(b,e)** have been published in Mahata et al. (2022a).

**Figure 8:**
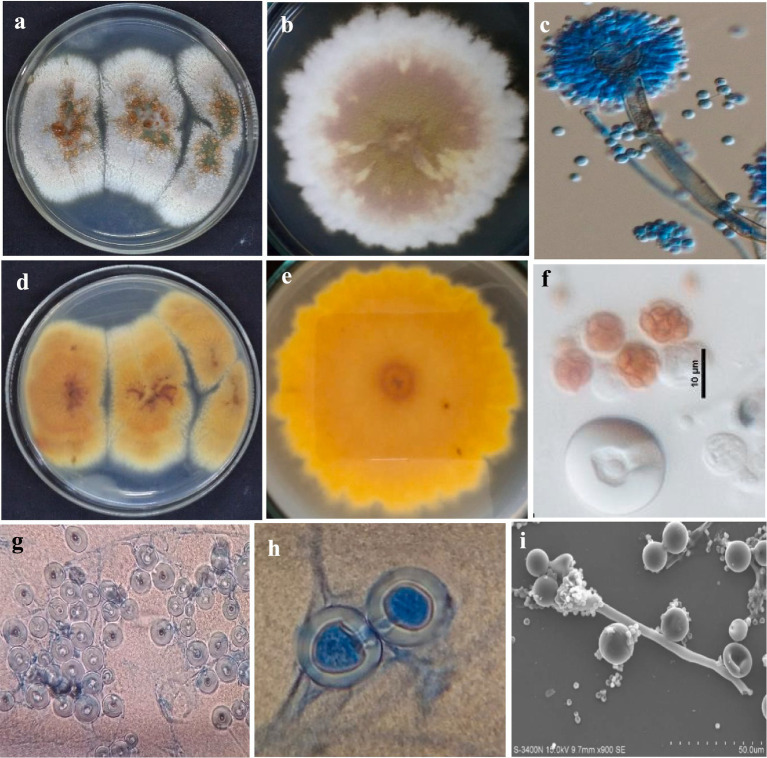
*Aspergillus nidulans* (Acc. No. MN791101) colonies, conidiophores, vesicles, conidia, asci, and Hülle cells. **(a,b,d,e)** Obverse and reverse views of the fungal isolate, incubated at 25 °C on PDA; **(c)** (DIC) Enlarged view of conidiophores; **(f)** (DIC) Hülle cells and ascospores in asci; **(g,h)** (CLM) Hülle cells; **(i)** (SEM) Mycelium and Hülle cell development; Scale bars: **(f)** = 10 μm; **(i)** = 50 μm. The figures **(b,c,e,f,i)** have been published in Mahata et al. (2022a).

**Figure 9:**
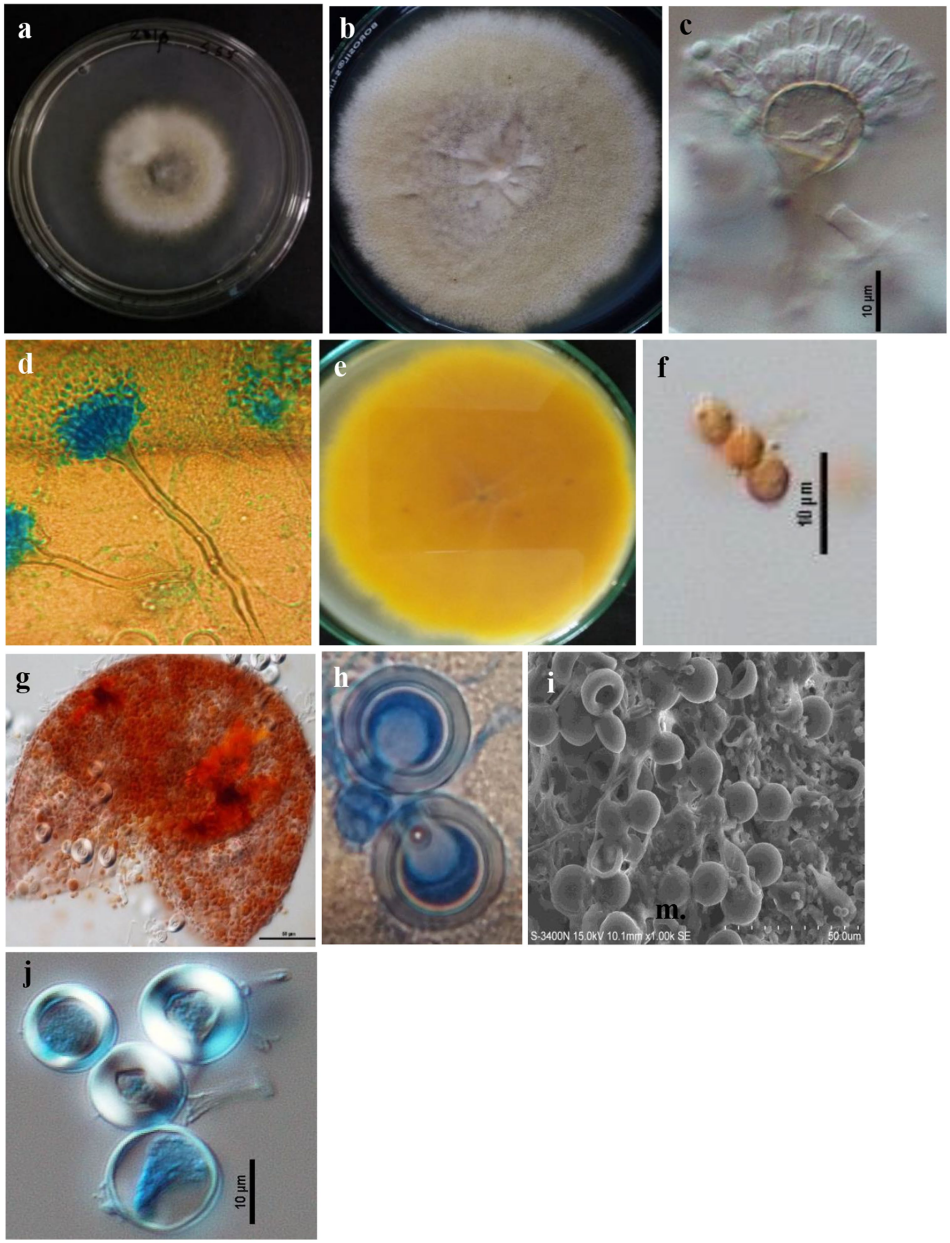
*Aspergillus quadrilineatus/ Emericella quadrilineata* (Acc. No. MN791105) colonies, conidiophores, vesicles, conidia, cleistothecia, ascospores, and Hülle cells. **(a,b,e)** Obverse and reverse views of the fungal isolate, incubated at 25 °C on PDA; **(c)** (DIC) smooth, sinuate conidiophores and short, columnar conidial heads with hemispherical vesicles, and conidia; **(d)** (CLM) conidiophores; **(f)** lenticular, smooth walled (unstained with cotton-blue) ascospores; **(g)** (DIC) ruptured cleistothecium; **(h)** (CLM) Hülle cells; **(i)** (SEM) Hülle cell development; **(j)** (DIC) Hülle cells. Scale bars: **(g,i)** = 50 μm; **(c,f,j)** = 10 μm. Figures (b–j), have been published in Mahata et al. (2022a).

**Figure 10:**
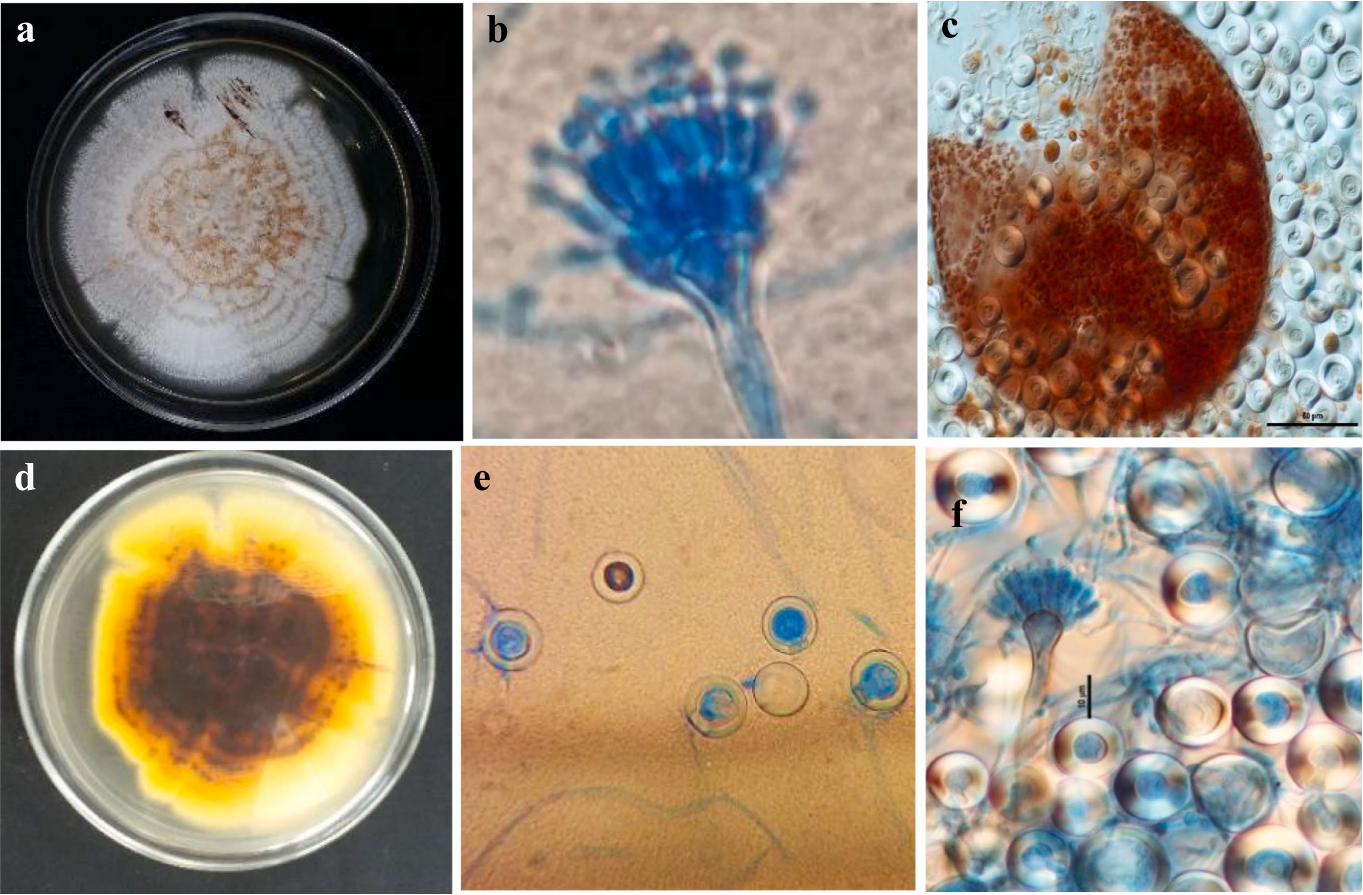
*Aspergillus latus* (Acc. No. MN791110) colonies, conidiophores, vesicles, sterigmata, conidia, cleistothecia, asci, ascospores, and Hülle cells. **(a,d)** Obverse and reverse view of the fungal isolate, incubated at 25 °C on PDA; **(b)** (CLM) conidiophore; **(c)** (DIC) the asci-containing cleistothecium, encased in Hülle cells, has ruptured; **(e)** (CLM) Hülle cells; **(f)** (DIC) Hülle cells. Scale bars: **(c)** = 50 μm; **(f)** = 10 μm. The figures **(c,f)** have been published in Mahata et al. (2022a).

**Figure 11:**
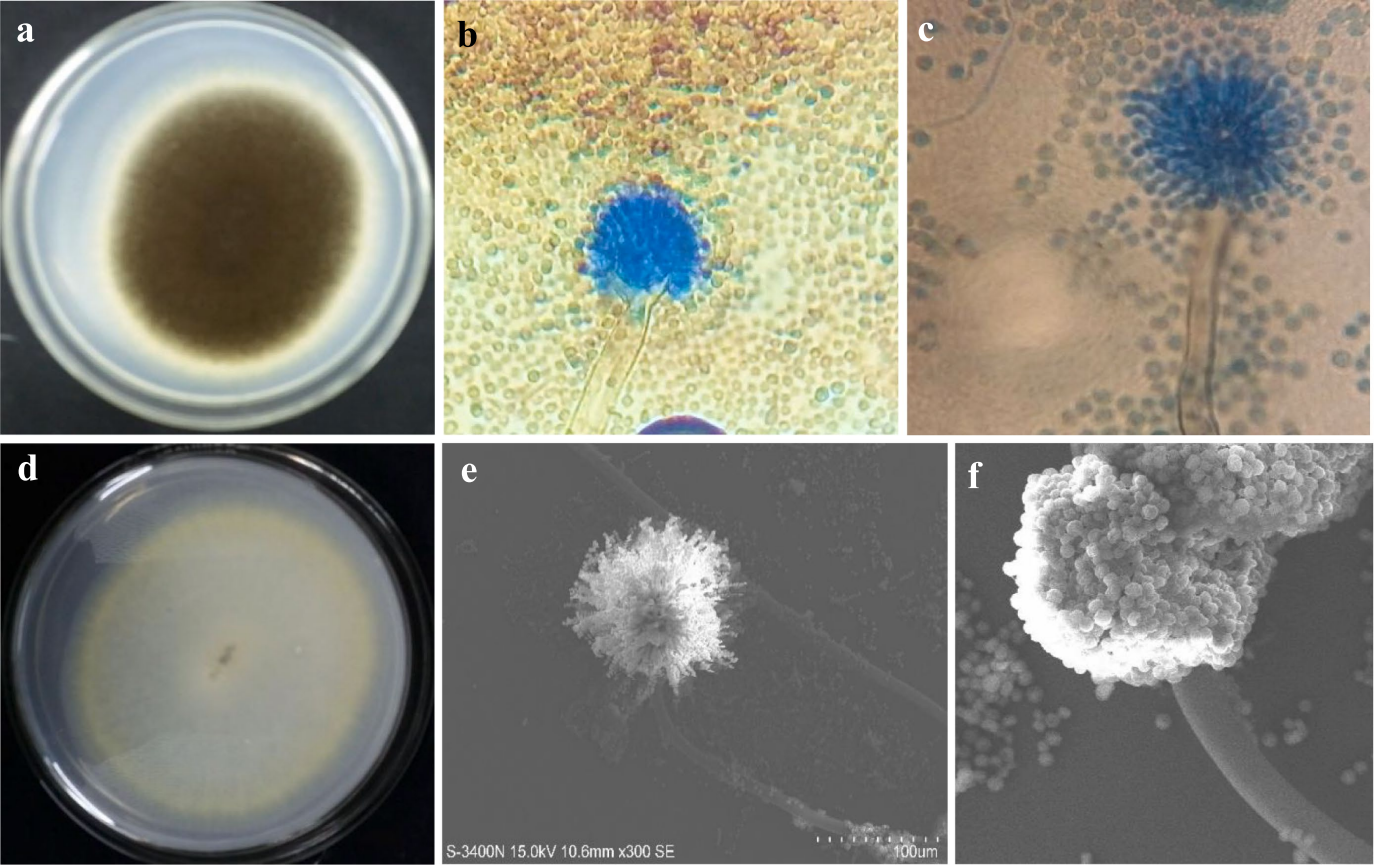
*Aspergillus awamori* (Acc. No. MN791114) colonies, conidiophores, vesicles, sterigmata, and conidia. **(a,d)** Obverse and reverse views of the fungal isolate, incubated at 25 °C on PDA medium; **(b,c)** (CLM) Conidiophores; **(e,f)** (SEM) Smooth conidiophores and large, globose conidial heads with globose vesicles, sterigmata, and chains of conidia. Scale bars: **(e,f)** 100 μm. The figures **(a,f)** have been published in Mahata et al. (2022a).

**Figure 12:**
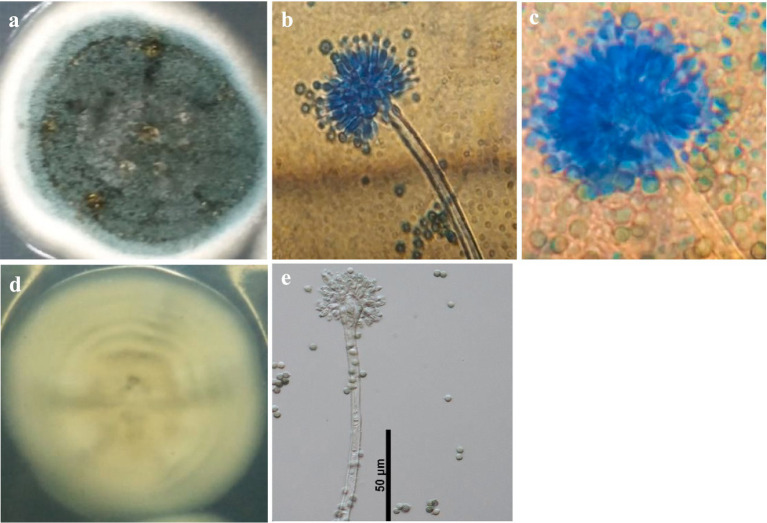
*Aspergillus sydowii* (Acc. No. MN298848) colonies, conidiophores, vesicles, sterigmata, and conidia. **(a,d)** Obverse and reverse views of the fungal isolate, incubated at 25 °C on PDA; **(b,c)** (CLM) Conidiophore and conidia; **(e)** (DIC) Smooth, sinuous conidiophores and hemispherical conidial heads with globose to elliptical vesicles, metulae, phialides, and conidia; Scale bars: **(e)** = 50 μm. The figures **(b,e)** have been published in Mahata et al. (2022a).

**Figure 13:**
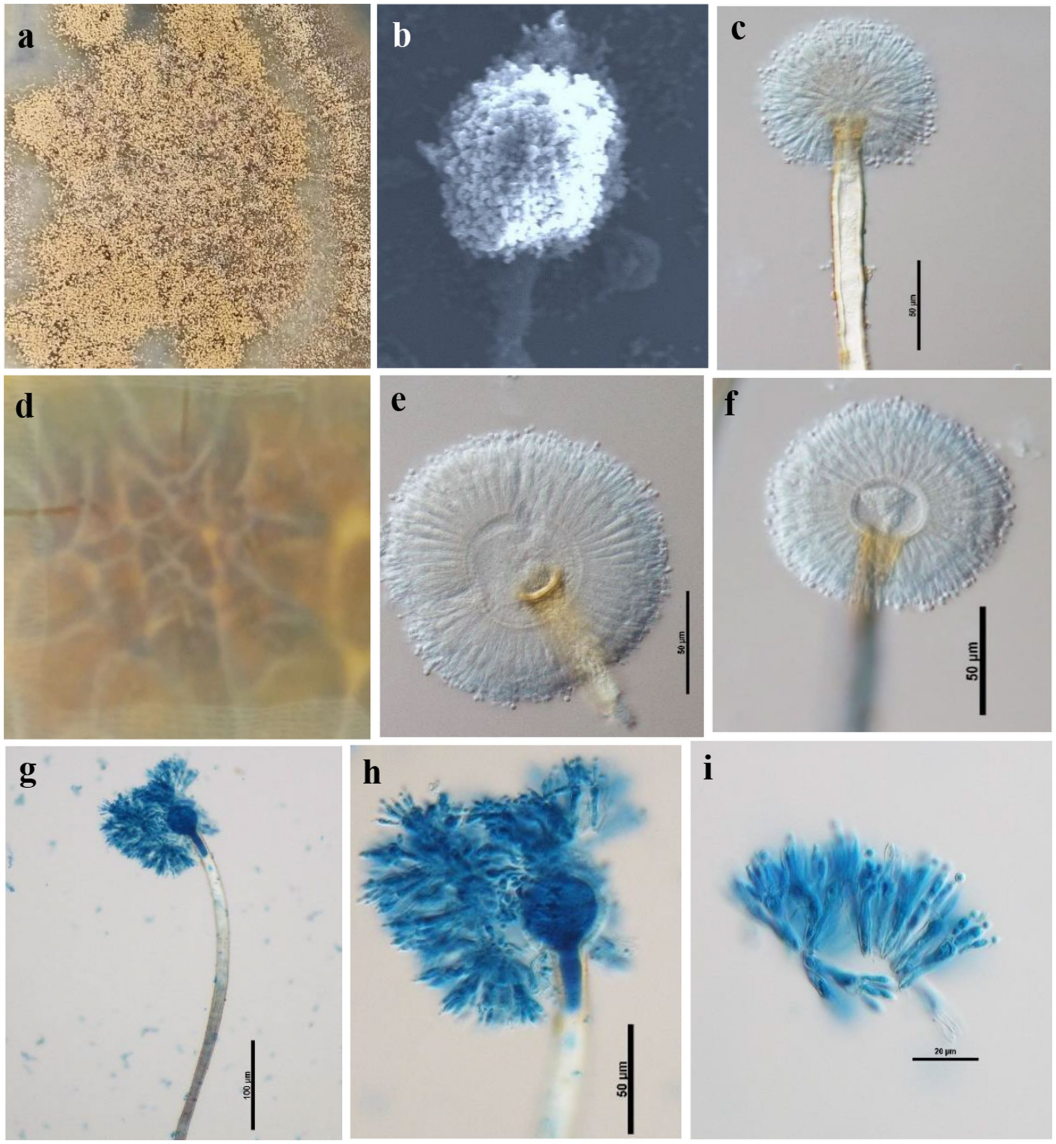
*Aspergillus* species (Acc. No. MN294688) colonies, conidiophores, vesicles, sterigmata, and conidia. **(a,d)** Obverse and reverse views of the fungal isolate, incubated at 25 °C on PDA; **(b)** (SEM) conidiophore; **(c,e–h)** (DIC) rough, pitted conidiophores, globose conidial heads with globose, thinner vesicles, sterigmata, and conidia; **(i)** (DIC) two series sterigmata and conidia. Scale bars: **(g)** = 100 μm; **(b,c,e,f,h)** = 50 μm; **(i)** = 20 μm.

**Figure 14:**
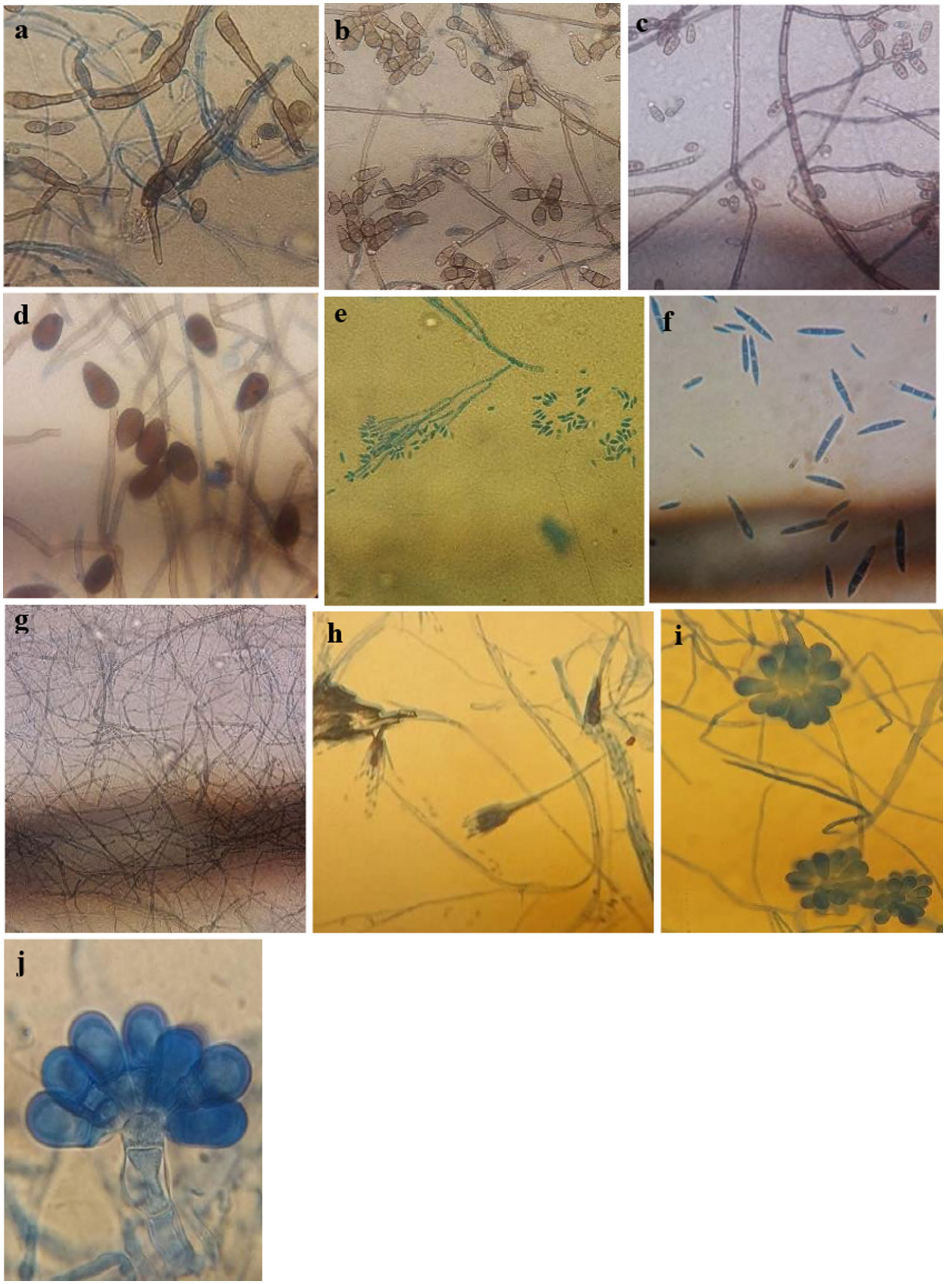
Photographs show hyphae, microconidia, macroconidia, conidiophores, and conidia captured using a compound light microscope (CLM). **(a)** Conidia of *A. alternata*; **(b)** *Curvularia*; **(c,d)** *Drechslera*; **(e)** microconidia of *Fusarium oxysporum*; **(f)** macroconidia of *F. verticillioides*; **(g)** *M. sterilia*; **(h)** conidia, sterigmata, and conidiophores *of Penicillium* sp.; **(i,j)** spores of *Trichothecium*.

Reliable species identification was achieved using standard taxonomic Fungal Keys and Manuals, reference Monographs and recent literature published in fungal taxonomy and identification methods (Gherbawy and El-Khallal, 2025). Fungal diversity in contaminated fennel seeds was evaluated through a thorough mycological investigation of seventy (70) distinct samples. Standard parameters which included cultural, macroscopic and microscopic analyses revealed the presence of twenty-five (25) different fungal species. The Figures 2–14 shows the pictographic and micrographic evidence of the representative fungal genera, which illustrate the primary diagnostic characteristics for genus-level identification.

### Quantitative studies of fungal colonization in *F. vulgare* to determine percent incidence and frequency of occurrence

#### High frequency of occurrence of fungi

Among the twenty-five (25) species recorded, *A. niger* was the most frequently encountered species (32.23%) followed by *Mycelia sterilia* (21.26%), a group of sterile mycelial and septate fungi that lacked identifiable asexual and/ or sexual structures. Other genera that were prevalent included *A. flavus* (11.62%), *Mucor* sp. (9.24%), *Penicillium* sp. (8.12%), and *Saccharomyces cerevisiae* (4.84%). Other fungi which occurred in descending order were *Neurospora crassa* (2.37%), *A. terreus* (2.6%), *A. nidulans* (1.64%), *Curvularia* sp. (1.03%) (Table 2).

**Table 2:** Prevalence groups of Fungi in all the seventy samples of *F. vulgare*- high, moderate, and low frequencies of occurrence.

**Genus/ Species**	**Prevalence percentage (%)**
High prevalence (>10%)	
*A. niger*	32.235
*M. sterilia*	21.266
*A. flavus*	11.628
Moderate prevalence (1–10%)	
*Mucor* sp.	9.248
*Penicillium* sp.	8.126
*S. cerevisiae*	4.848
*A. terreus*	2.604
*N. crassa*	2.379
*A. nidulans*	1.646
*Curvularia* sp.	1.032
Low prevalence (<1%)	
*A. tamarii*	0.972
*Aspergillus* sp.	0.957
*E. quadrilineata*	0.448
*A. fumigatus*	0.434
*A. latus*	0.404
*A. aureoterreus*	0.374
*A. awamori*	0.374
*A. brasiliensis*	0.299
*A. alternata*	0.284
*Drechslera* sp.	0.149
*F. oxysporum*	0.149
*A. ochraceous*	0.059
*A. sydowii*	0.029
*F. verticillioides*	0.029
*T. roseum*	0.014

#### Low frequency of occurrence of fungi

*A. alternata* (0.28%), *Drechslera* sp. (0.14%), *Fusarium oxysporum* (0.14%), *A. ochraceous* (0.05%), *A. sydowii* (0.02%), *F. verticillioides* (0.02%), and *Trichothecium* sp. (0.01%) were classified of fungi with low frequency (Table 2).

#### Low frequency of occurrence of *Aspergilli*

*Aspergilli* with low frequency of occurrence were recorded with *A. tamarii* (0.97%), *Aspergillus* sp. (0.95%), *Emericella quadrilineata* (0.44%), *A. fumigatus* (0.43%), *A. latus* (0.40%), *A. aureoterreus*, *A. awamori* (0.37%), and *A. brasiliensis* (0.29%) (Table 2).

### Cultural, microscopic, and molecular detection *Aspergilli*

Species-level identification was achieved with eleven (11) *Aspergilli* (Figures 3–13), isolated and identified from fennel seed samples, the details of which are presented in Table 1. In order to perform conclusive analyses, barcoding using the universal fungal barcode, namely ITS provided valuable species-level identification. The use of a functional genetic marker namely β-tubulin served additionally in species-level identification, enabling multi-gene phylogeny and determination of phylogenetic relationships (Mahata et al., 2022a) with previously described species deposited at the National Centre for Biotechnological Information (NCBI). The anamorphic and telomorphic names of the *Aspergilli* from the *Nidulantes* Section are also presented Table 1. The sample-wise dominant species and the levels of prevalence- very high, high, moderate and low is outlined in Table 3.

**Table 3:** Percent incidence (%) of fungi from different states in India- very high, high, moderate and low levels.

Infection category	Percent infection and range (%)	**Percent incidence of fungi in different states of India**									
		**Pondicherry (30)**		**West Bengal (12)**		**New Delhi (11)**		**Uttar Pradesh (11)**		**Rajasthan (06)**	
		Numbers	Frequency (%)	Numbers	Frequency (%)	Numbers	Frequency (%)	Numbers	Frequency (%)	Numbers	Frequency (%)
Very High	100	18	60.0	7	58.33	9	81.8	8	72.7	5	83.3
High	90–99	7	23.3	4	33.33	2	18.2	3	27.3	1	16.7
Moderate	70–89	3	10.0	1	8.33	0	0.0	0	0.0	0	0.0
Low	<70	2	6.7	0	0.00	0	0.0	0	0.0	0	0.0

Total number of samples from each state is mentioned in the bracket.

## Discussion

The current analyses offer new perspectives on the fungal contamination in fennel seeds, a significant research topic that has not been previously documented in the scientific literature (Sharma et al., 2022), especially from India. The findings of this study are of enormous importance to public health and food safety, particularly in India, due to the medicinal significance and consumption of fennel (Kumar and Tripathi, 2021) in humans. This investigation covered several study locations in India, including both, fennel-producing and non-producing states, thereby facilitating a more comprehensive study of the post-harvest contamination and the possible climatic influences on fungal growth (Ramesh et al., 2020) and proliferation.

A substantial number of fennel seeds were collected (30 out of 70 samples) from Pondicherry, although it is a non-producing/ non-cultivating region. The rationale being that the city being the study location, offered logistical advantages. Further, the spice is commonly and frequently used in most of their culinary preparations. Laboratory and mycological studies, emphasize the possibility of post-harvest contamination during storage or transit (Das et al., 2021). For xerophilic and thermotolerant species, the mild winter during the January (2017) months (26 °C and 77% RH), transitioning to a slightly drier and hot weather by July (32 °C, 70% RH), provides favorable conditions for fungal growth (Rana et al., 2022) thereby jeopardizing the storage quality of grains and seeds.

Another non-producing state, New Delhi was also included as one of our study locations. A favorable temperature and relative humidity (31 °C, 77% RH) is suitable for fungal growth during storage (Verma and Sinha, 2021). The intentional inclusion of non-producing states in the sampling procedure was aimed at assessing the extent of post-harvest contamination. The presence of fungal genera in these samples is clearly indicative of contamination, which occur during post-harvest storage, due to improper and unscientific storage methods, rather than at the field-level (Das et al., 2021).

In contrast, the contamination profiles from the seed samples of fennel-producing states namely West Bengal, Rajasthan, and Uttar Pradesh were notably different displaying lower contamination levels as evident in our isolation studies. The fennel seeds (12 samples) were obtained from West Bengal under cooler and highly humid conditions (17–20 °C, 95% RH) (Nair and Joshi, 2023). Lower contamination levels may be attributed to shorter storage durations before sampling, temperature and relative humidity which are critical factors in preventing the proliferation of fungi resulting in contamination (Verma and Sinha, 2021). However, they may remain in warehouses for extended periods under suboptimal conditions (Li et al., 2026).

Samples collected in September 2017 from Uttar Pradesh and Rajasthan were comparable to those from West Bengal. With an ambient temperature of 30 °C and relative humidity of 78%. Such moderately warm and humid conditions support ample growth of numerous mycotoxigenic fungi (Jeswal and Kumar, 2015), as is evident from our samples (Tables 4, 5) which displayed infection rates between 95 and 100%.

**Table 4:** Frequencies of occurrence expressed as percent incidence of individual fungal genera/ species isolated from Uttar Pradesh fennel seeds (11 samples).

Sample ID	Dominant species	Percent incidence (%)	Other genera	Percent incidence (%)
S60-FOEV60	*Mucor* sp.	37.37	*A. niger*	25.25
			*S. cerevisiae*	13.13
			*A. flavus*	12.12
			*M. sterilia*	10.1
			*Penicillium* sp.	2.02
S61-FOEV61	*Penicillium* sp.	28	*A. niger*	21
			*A. nidulans*	15
			*A. flavus*	12
			*M. sterilia*	11
			*Curvularia* sp.	6
			*S. cerevisiae*	6
			*A. sydowii*	1
S62-FOEV62	*A. niger*	39	*M. sterilia*	26
			*A. terreus*	19
			*A. flavus*	9
			*N. crassa*	4
			*A. nidulans*	3
S63-FOEV63	*A. flavus*	54	*A. niger*	21
			*Mucor* sp.	18
			*M. sterilia*	4
			*E. quadrilineata*	2
S64-FOEV64	*A. niger*	50	*S. cerevisiae*	11
			*A. terreus*	9
			*M. sterilia*	8
			*A. flavus*	7
			*A. alternata*	3
			*A. nidulans*	3
			*Penicillium* sp.	3
			*A. tamarii*	2
			*Curvularia* sp.	2
			*Mucor* sp.	2
S65-FOEV65	*A. niger*	42	*Mucor* sp.	15
			*M. sterilia*	12
			*A. flavus*	10
			*A. nidulans*	7
			*Curvularia* sp.	7
			*S. cerevisiae*	6
			*Penicillium* sp.	1
S66-FOEV66	*A. niger*	35	*A. flavus*	20
			*M. sterilia*	17
			*Penicillium* sp.	10
			*S. cerevisiae*	10
			*A. nidulans*	7
			*Drechslera* sp.	1
S67-FOEV67	*M. sterilia*	26.31	*N. crassa*	20
			*A. terreus*	16.84
			*Penicillium* sp.	16.84
			*A. niger*	10.52
			*S. cerevisiae*	5.26
			*A. flavus*	2.1
			*Curvularia* sp.	2.1
S68-FOEV68	*Mucor* sp.	32	*M. sterilia*	30
			*A. niger*	22
			*A. flavus*	11
			*S. cerevisiae*	4
			*Penicillium* sp.	1
S69-FOEV69	*A. niger*	20.61	*M. sterilia*	17.52
			*A. flavus*	16.49
			*Curvularia* sp.	12.37
			*S. cerevisiae*	11.34
			*Penicillium* sp.	8.24
			*A. terreus*	7.21
			*F. oxysporum*	4.12
			*A. alternata*	1.03
			*A. fumigatus*	1.03
S70-FOEV70	*A. niger*	31	*A. flavus*	18
			*M. sterilia*	18
			*S. cerevisiae*	14
			*Penicillium* sp.	7
			*A. latus*	4
			*Curvularia* sp.	4
			*A. terreus*	2
			*Drechslera* sp.	2

**Table 5:** Frequencies of occurrence expressed as percent incidence of individual fungal genera/ species isolated from Rajasthan fennel seeds (06 samples).

Sample ID	Dominant species	Percent incidence (%)	Other genera	Percent incidence (%)
S54-FOEV54	*A. niger*	50	*A. flavus*	9
			*A. nidulans*	7
			*A. terreus*	7
			*Curvularia* sp.	6
			*A. tamarii*	5
			*M. sterilia*	4
			*Penicillium* sp.	4
			*S. cerevisiae*	3
			*A. awamori*	2
			*E. quadrilineata*	2
			*Drechslera* sp.	1
S55-FOEV55	*A. niger*	55	*A. flavus*	21
			*A. terreus*	7
			*A. nidulans*	5
			*Penicillium* sp.	5
			*S. cerevisiae*	4
			*M. sterilia*	2
			*Drechslera* sp.	1
S56-FOEV56	*A. niger*	66	*Penicillium* sp.	13
			*A. flavus*	12
			*M. sterilia*	8
			*A. fumigatus*	1
S57-FOEV57	*A. niger*	36.66	*Mucor* sp.	21.11
			*A. flavus*	15.55
			*A. terreus*	15.55
			*A. nidulans*	3.33
			*M. sterilia*	3.33
			*Penicillium* sp.	2.22
			*A. fumigatus*	1.11
			*A. tamarii*	1.11
S58-FOEV58	*A. niger*	58	*A. flavus*	11
			*Aspergillus* sp.	9
			*M. sterilia*	9
			*A. nidulans*	6
			*Penicillium* sp.	6
			*A. fumigatus*	1
S59-FOEV59	*A. niger*	27	*M. sterilia*	26
			*A. flavus*	16
			*S. cerevisiae*	10
			*A. terreus*	8
			*Curvularia* sp.	8
			*Penicillium* sp.	3
			*A. fumigatus*	2

Sample-wise mycological analysis of *F. vulgare* seed samples, collected from five states (Puducherry, West Bengal, New Delhi, Uttar Pradesh, and Rajasthan) (Figure 1). Using macromorphological and micromorphological characterization revealed rich and diverse fungal communities. A total of twenty-five (25) fungal species were isolated and identified from 70 seed samples that were analyzed using conventional techniques (Table 2). The macroscopic characterization included key parameters like colony morphology, shape, size, pigmentation, surface characteristics, margin morphologies, and radial growth rate on PDA medium. These parameters were the initial steps adopted in the identification process as outlined in standard identification protocols, which established the essential basis for the fungal taxonomy. Compound light microscopy (CLM), scanning electron microscopy (SEM), and differential interference contrast microscopy (DIC) were implemented to analyze micromorphological characteristics (Mahata et al., 2022a; Mahata et al., 2022b). The nature of conidiophores, type, arrangement and septation of conidia, vesicles, phialides, presence or absence of specialized structures such as Cleistothecia (specific for *Aspergillus* species), Hulle cells, and chlamydospores (specific for *Fusarium* species) were all critical diagnostic characteristics in morphological identification (Figures 3–14). These characteristics were especially advantageous in the identification of closely related species within genera such as *Aspergillus*, *Penicillium*, and *Fusarium* species. It has been advantageous to include representative photographic documentation of fungal colonies and their microscopic characteristics in the micrographs which clearly depict the sporulating structures, conidial ornamentation (if any), and obvious morphological differences among taxa, along with molecular data for 11 selected *Aspergillus* species, which could serve as essential and useful information for researchers working in similar areas of specialization.

A diverse array of fungal species with varying levels of prevalence was identified through a thorough mycological analysis of the total number of seventy (70) samples. The individual fungal isolates were divided into three categories according to their relative abundance: high (>10%), moderate (1–10%), and low (<1%) prevalence. *A. niger* was the most prevalent fungus with a frequency of 32.235%, followed by *M. sterilia*, a non-sporulating morphotype, which accounted for 21.266%. The prevalence of *A. flavus* was also noticeable in this group, with a value of 11.628%. The dominance of these three species in the sampled fennel seeds was evident, as they constituted over 65% of the total fungal community that was observed (Table 2). A more variegated assemblage was present in the moderate prevalence group (1–10%). The yeast *S. cerevisiae* (4.848%) was followed by *Mucor* sp. (9.248%) and *Penicillium* sp. (8.126%) in descending order. Two *Aspergillus* species were observed, namely as *A. terreus* (2.604%) and *A. nidulans* (1.646%). *N. crassa* (2.379%) and *Curvularia* sp. (1.032%) were also moderately prevalent (Table 2). A significant number of fungi had a low prevalence (<1%). The frequencies within this cohort were that of *A. tamarii* (0.972%) and an unidentified *Aspergillus* species (0.957%). *E. quadrilineata* (0.448%), *A. fumigatus* (0.434%), *A. latus* (0.404%), and *A. aureoterreus* (0.374%) were additional rare isolates. Other *Aspergillus* species, including *A. awamori*, *A. brasiliensis*, *A. ochraceous*, and *A. sydowii*, were isolated, though at very low frequencies, ranging from 0.299 to 0.029%. Furthermore, infrequent genera, including *A. alternata* (0.284%), *Drechslera* sp. (0.149%), *F. oxysporum* (0.149%), *F. verticillioides* (0.029%), and *Trichothecium* sp. (0.014%), were identified (Table 2).

Numerous regional variations were observed in the percentage of fungal infections throughout the several states of India. With 60.0% of samples falling into the very high infection level of 100 and 23.3% falling into the high infection level of 90–99%, Pondicherry (n = 30) had the most various types of sicknesses. Despite the short sample size, the fungus was severe in Rajasthan (n = 6), where 83.3% of the participants had a very high infection rate. There were a disproportionately large number of cases in New Delhi (n = 11), with 81.8% of samples rated as extremely high and 18.2% as high. A large frequency of fungal infections was shown by the 72.7% very high infection and 27.3% high infection rates in Uttar Pradesh (n = 11). There was a small fraction (8.33%) classed as moderate infection, and 58.33% as extremely high infection in West Bengal (n = 12). Out of all the states surveyed, only Pondicherry (10.0%) and West Bengal (8.33%) had moderate infection (70–89%); the states in the north and west did not have it. Only in Pondicherry (6.7% infection rate) was a low infection rate (less than 70%). It is evident that there is a significant amount of fungal contamination, as the very high infection category is the most common across all states (Table 3). This increased rate could be caused by factors such as high humidity, sudden changes in temperature, or improper storage. Based on these findings, India has to enhance its post-harvest procedures for handling, storing, and monitoring fungi in various agro-climatic regions.

In samples collected from Pondicherry, the fungal colonization of 30 seed samples (FOEV1-FOEV15; FOEV28-FOEV42) was studied (Table 6), exhibiting a diverse community structure with infection rates ranging from 61 to 100%. Several fungal species were consistently detected across multiple samples, with *A. niger*, *M. sterilia*, *Penicillium* sp., *Mucor* sp. and *S. cerevisiae* being among the most dominant species. In 15 samples (FOEV3-5, FOEV8, FOEV9, FOEV11-14, FOEV28, FOEV31-32, FOEV34, FOEV38, FOEV41, and FOEV42), *A. niger* was the most frequently dominant species, heading the fungal community, FOEV12 (73%) and FOEV14 (71%) exhibiting the highest levels of dominance. *M. sterilia*, *A. flavus*, *Penicillium* sp., and *A. aureoterreus* were among the other species that frequently co-occurred with *A. niger*. *M. sterilia* occurred as a major candidate of the fungal biota, predominating in samples FOEV7, FOEV10, FOEV15, FOEV30, FOEV33, and FOEV37. *M*. *sterilia* being a non-taxonomic group of fungi, mostly belong to Ascomycetes and Basidiomycetes. This group of fungi do not display sporulation due to several biotic and abiotic factors. The presence of *M*. *sterilia* in fennel seed samples cannot be ignored in such mycological studies because they can be agents of plant pathogenesis affecting cereals and pulses (Deepika et al., 2020). Their dominance was mostly evident in FOEV7 (83%) and FOEV10 (60.65%), being present in conjunction with *A. niger* and other *Aspergillus* species. *Penicillium* sp. was the major species in FOEV1, FOEV2, and FOEV6, with a prevalence of 62.24% in FOEV6. The intense localized colonization of *Mucor* sp. was indicated by its significant dominance in FOEV29 (32%), FOEV35 (35%), and *F*OEV39 (59%). The frequency of occurrence in other samples was low (FOEV2, FOEV4, and FOEV14). *S. cerevisiae* was the main yeast species in FOEV36 (45.83%) and FOEV40 (72.72%), with low frequencies in samples FOEV2, FOEV4, FOEV39, FOEV41, and FOEV42. *N. crassa*, *Fusarium* sp., *Curvularia* sp., *Drechslera* sp., *Trichothecium* sp., *E. quadrilineata*, and rare *Aspergilli* (*A. tamarii*, *A. latus*, *A. awamori*) were among the filamentous fungi that contributed to the diverse fungal community. In summary, the findings suggest that *A. niger* and *M. sterilia* were the most frequently encountered and dominant species in the samples of Pondicherry, frequently co-occurring with other common vegetative and pathogenic fungi (Table 6).

**Table 6:** Frequencies of occurrence expressed as percent incidence of individual fungal genera/ species isolated from Pondicherry fennel seeds (30 samples).

Sample ID	Dominant species	Percent incidence (%)	Other genera	Percent incidence (%)
S1-FOEV1	*Penicillium* sp.	49	*M. sterilia*	19
			*A. niger*	13
			*A. flavus*	12
			*A. aureoterreus*	3
			*A. fumigatus*	2
			*A. alternata*	2
S2-FOEV2	*Penicillium* sp.	35.35	*Mucor* sp.	25.25
			*M. sterilia*	25.25
			*A. niger*	10.1
			*A. fumigatus*	3.03
			*S. cerevisiae*	1.01
S3-FOEV3	*A. niger*	50.52	*Penicillium* sp.	34.73
			*M. sterilia*	7.36
			*A. awamori*	4.21
			*A. flavus*	3.15
S4-FOEV4	*A. niger*	50	*M. sterilia*	30
			*A. flavus*	5.71
			*S. cerevisiae*	5.71
			*Mucor* sp.	4.28
			*A. aureoterreus*	1.42
			*Penicillium* sp.	1.42
			*Trichothecium* sp.	1.42
S5-FOEV5	*A. niger*	47.82	*Penicillium* sp.	18.84
			*Curvularia* sp.	15.94
			*M. sterilia*	14.49
			*A. flavus*	1.44
			*F. oxysporum*	1.44
S6-FOEV6	*Penicillium* sp.	62.24	*A. niger*	23.46
			*M. sterilia*	14.28
S7-FOEV7	*M. sterilia*	83	*A. niger*	11
			*A. terreus*	4
			*A. aureoterreus*	1
			*F. oxysporum*	1
S8-FOEV8	*A. niger*	60	*M. sterilia*	20
			*A. flavus*	16
			*A. alternata*	4
S9-FOEV9	*A. niger*	51	*M. sterilia*	33
			*A. flavus*	14
			*A. aureoterreus*	2
S30-FOEV30	*M. sterilia*	48.48	*A. tamarii*	15.15
			*E. quadrilineata*	11.11
			*Aspergillus* sp.	10.1
			*A. niger*	5.05
			*S. cerevisiae*	5.05
			*A. flavus*	2.02
			*A. terreus*	2.02
			*A. latus*	1.01
S31-FOEV31	*A. niger*	58	*M. sterilia*	12
			*A. flavus*	9
			*Aspergillus* sp.	9
			*A. nidulans*	6
			*A. alternata*	3
			*A. tamarii*	2
			*Curvularia* sp.	1
S32-FOEV32	*A. niger*	55	*A. latus*	17
			*A. tamarii*	15
			*A. flavus*	10
			*Drechslera* sp.	2
			*E. quadrlineata*	1
S33-FOEV33	*M. sterilia*	28.15	*A. niger*	26.04
			*A. flavus*	11.45
			*N. crassa*	9.37
			*Penicillium* sp.	9.37
			*Aspergillus* sp.	6.25
			*E. quadrlineata*	4.16
			*A. tamarii*	2.08
			*A. latus*	1.04
			*A. nidulans*	1.04
			*A. sydowii*	1.04
S34-FOEV34	*A. niger*	66	*Penicillium* sp.	13
			*M. sterilia*	12
			*A. flavus*	3
			*A. nidulans*	3
			*A. tamarii*	2
			*A. awamori*	1
S35-FOEV35	*Mucor* sp.	35	*M. sterilia*	21
			*A. niger*	13
			*A. flavus*	8
			*A. nidulans*	7
			*A. tamarii*	6
			*Penicillium* sp.	6
			*S. cerevisiae*	3
			*A. awamori*	1
S36-FOEV36	*S. cerevisiae*	45.83	*A. flavus*	25
			*Aspergillus* sp.	20.85
			*A. tamarii*	8.33
S37-FOEV37	*M. sterilia*	50.63	*A. tamarii*	12.65
			*Aspergillus* sp.	12.65
			*A. nidulans*	10.12
			*A. niger*	7.59
			*A. fumigatus*	2.53
			*A. latus*	2.53
			*F. verticillioides*	1.26
S38-FOEV38	*A. niger*	36	*M. sterilia*	26.66
			*A. flavus*	21.33
			*A. terreus*	5.33
			*A. tamarii*	4
			*Aspergillus* sp.	4
			*A. fumigatus*	1.33
			*Penicillium* sp.	1.33
S39-FOEV39	*Mucor* sp.	59	*M. sterilia*	17
			*S. cerevisiae*	16
			*A. flavus*	5
			*A. niger*	2
			*A. fumigatus*	1
S40-FOEV40	*S. cerevisiae*	72.72	*M. sterilia*	24.24
			*A. terreus*	3.03
S41-FOEV41	*A. niger*	38.55	*M. sterilia*	20.48
			*A. terreus*	16.86
			*A. flavus*	9.63
			*E. quadrilineata*	4.81
			*S. cerevisiae*	4.81
			*Penicillium* sp.	3.61
S42-FOEV42	*A. niger*	54.73	*Mucor* sp.	14.73
			*M. sterilia*	11.57
			*A. terreus*	8.42
			*N. crassa*	4.21
			*A. flavus*	3.15
			*S. cerevisiae*	2.1
			*Penicillium* sp.	1.05

In order determine the diversity of species and the rates of fungal infection from West Bengal (Table 7), 12 seed samples of *F. vulgare* were subjected to mycological examination. The seeds exhibited fungal colonization, as evidenced by high infection rates, which ranged from 82 to 100%. *M. sterilia* was the most dominant species as observed in samples collected from Pondicherry, leading the infection profile in seven samples (FOEV16, FOEV17, FOEV20-23, and FOEV25). The maximum occurrence was observed in FOEV20 (48.45%) and FOEV23 (45%). Its extensive prevalence was also evident in the detection of it as a co-dominant species in numerous other samples. The fungal burden in other samples has been described in Table 7.

**Table 7:** Frequencies of occurrence expressed as percent incidence of individual fungal genera/ species isolated from West Bengal fennel seeds (12 samples).

Sample ID	Dominant species	Percent incidence (%)	Other genera	Percent incidence (%)
S16-FOEV16	*M. sterilia*	43.43	*A. niger*	34.34
			*A. flavus*	13.13
			*A. aureoterreus*	4.04
			*A. ochraceous*	4.04
			*A. terreus*	1.01
S17-FOEV17	*M. sterilia*	31	*A. niger*	29
			*Mucor* sp.	29
			*A. brasiliensis*	7
			*A. flavus*	3
			*S. cerevisiae*	1
S18-FOEV18	*S. cerevisiae*	65	*M. sterilia*	23
			*A. terreus*	7
			*F. oxysporum*	3
			*A. niger*	2
S19-FOEV19	*Mucor* sp.	35	*A. niger*	21
			*M. sterilia*	20
			*A. flavus*	10
			*S. cerevisiae*	7
			*A. brasiliensis*	5
			*A. awamori*	2
S20-FOEV20	*M. sterilia*	48.45	*A. flavus*	21.64
			*A. niger*	21.64
			*A. aureoterreus*	3.09
			*A. terreus*	3.09
			*A. awamori*	2.06
S21-FOEV21	*M. sterilia*	42	*N. crassa*	20
			*A. flavus*	17
			*A. niger*	14
			*A. brasiliensis*	3
			*A. terreus*	3
			*Curvularia* sp.	1
S22-FOEV22	*M. sterilia*	37	*A. niger*	29
			*A. flavus*	23
			*Curvularia* sp.	7
			*A. aureoterreus*	3
			*A. brasiliensis*	1
S23-FOEV23	*M. sterilia*	45	*A. niger*	29
			*A. flavus*	21
			*A. aureoterreus*	3
			*A. terreus*	1
			*Drechslera* sp.	1
S24-FOEV24	*Penicillium* sp.	59.79	*M. sterilia*	36.08
			*A. alternata*	2.06
			*A. brasiliensis*	1.03
			*Aspergillus* sp.	1.03
S25-FOEV25	*M. sterilia*	36.58	*Penicillium* sp.	28.04
			*A. flavus*	17.07
			*A. niger*	17.07
			*Curvularia* sp.	1.21
S26-FOEV26	*Mucor* sp.	42.1	*Penicillium* sp.	33.68
			*A. niger*	14.73
			*A. brasiliensis*	3.15
			*M. sterilia*	3.15
			*A. awamori*	1.05
			*A. flavus*	1.05
			*Aspergillus* sp.	1.05
S27-FOEV27	*Mucor* sp.	43	*M. sterilia*	29
			*Penicillium* sp.	14
			*A. nidulans*	10
			*A. fumigatus*	3
			*A. awamori*	1

Fungal enumeration methods employed to evaluate the contamination of 11 seed samples of *F. vulgare* from New Delhi (Table 8) helped us document fungal contaminants namely *A. niger*, *A. flavus*, and *Mucor* sp. The evidence of substantial fungal colonization across all samples was confirmed by the remarkably high total infection rate, which ranged from 92 to 100%. The most commonly detected dominant species was *A. niger*, which was present in five samples (FOEV46, FOEV47, FOEV49, FOEV50, FOEV52, FOEV53). The maximum levels were observed in samples FOEV46 (69%) and FOEV53 (63%). *A. flavus* was isolated from FOEV45 (37.11%) and FOEV48 (59%) and is of specific human and animal health concern because of its ability to produce Aflatoxins (AFs). *Mucor* sp. was also highly dominant in *FOEV44* (82%), with some other genera. *Penicillium* sp., *S. cerevisiae*, *A. nidulans*, and *N. crassa* were significantly abundant in FOEV51 (28%), and highly prevalent in FOEV45 and FOEV50. These results suggest that the fungal microbiota associated with *F. vulgare* seeds in New Delhi was both diverse (Table 8) and dense (Table 3).

**Table 8:** Frequencies of occurrence expressed as percent incidence of individual fungal genera/ species isolated from New Delhi fennel seeds (11 samples).

Sample ID	Dominant species	Percent incidence (%)	Other genera	Percent incidence (%)
S43-FOEV43	*M. sterilia*	64	*A. terreus*	17
			*A. flavus*	9
			*Mucor* sp.	3
			*Penicillium* sp.	3
			*S. cerevisiae*	2
			*A. fumigatus*	1
			*E. quadrilineata*	1
S44-FOEV44	*Mucor* sp.	82	*M. sterilia*	8
			*A. flavus*	5
			*A. fumigatus*	4
			*A. niger*	1
S45-FOEV45	*A. flavus*	37.11	*Mucor* sp.	16.49
			*M. sterilia*	14.43
			*A. niger*	13.4
			*N. crassa*	12.37
			*A. terreus*	4.12
			*Penicillium* sp.	1.03
			*S. cerevisiae*	1.03
S46-FOEV46	*A. niger*	69	*M. sterilia*	18
			*Mucor* sp.	13
S47-FOEV47	*A. niger*	39.13	*N. crassa*	23.91
			*A. flavus*	20.65
			*Mucor* sp.	5.43
			*S. cerevisiae*	5.43
			*A. latus*	2.17
			*M. sterilia*	2.17
			*A. alternata*	1.08
S48-FOEV48	*A. flavus*	59	*A. niger*	24
			*Mucor* sp.	9
			*A. nidulans*	5
			*Penicillium* sp.	2
			*A. alternata*	1
S47-FOEV49	*A. niger*	25	*A. flavus*	21
			*Penicillium* sp.	19
			*S. cerevisiae*	15
			*M. sterilia*	11
			*Mucor* sp.	9
S50-FOEV50	*A. niger*	38	*A. flavus*	22
			*N. crassa*	16
			*Penicillium* sp.	13
			*S. cerevisiae*	8
			*A. nidulans*	3
S51-FOEV51	*N. crassa*	28	*A. niger*	24
			*Mucor* sp.	20
			*A. flavus*	16
			*M. sterilia*	7
			*A. terreus*	4
			*A. alternata*	1
S52-FOEV52	*A. niger*	36	*A. nidulans*	18
			*A. flavus*	14
			*M. sterilia*	12
			*Aspergillus* sp.	7
			*Penicillium* sp.	6
			*E. quadrilineata*	5
			*Mucor* sp.	2
S53-FOEV53	*A. niger*	63	*A. flavus*	15
			*Penicillium* sp.	10
			*S. cerevisiae*	5
			*Mucor* sp.	4
			*A. nidulans*	3

The fungal infection rates of eleven (11) *F. vulgare* seed samples from Uttar Pradesh were consistently high, with levels ranging from 95 to 100%, as revealed by mycofloral analysis. The results indicated that the occurrence patterns, were highly diverse and recorded *A. niger* as the most prevalent and dominant fungal species (50%) in FOEV64, and in five other samples (FOEV62, FOEV65, FOEV66, FOEV69, FOEV70). It also appeared frequently in almost all samples tested from Uttar Pradesh, which further substantiated its significant ecological prevalence in the region. Following in abundance, *A. flavus* was documented in FOEV63 (54%). Two samples (FOEV60 and FOEV68) were dominated by *Mucor* sp., occurring at frequencies of 37.37 and 32%, respectively. *M. sterilia* was the predominant species in FOEV67 (26.31%) and was a significant component of the fungal diversity dataset. *Penicillium* sp. was frequently observed and exhibited incidence in FOEV61 (28%). *S. cerevisiae*, *A. nidulans*, *A. terreus*, *Curvularia* sp., *Drechslera* sp., and *N. crassa* were the other species isolated. The seed-associated mycofloral profile also highlighted the detection of *F. oxysporum*, *A. latus*, *A. tamarii*, *A. fumigatus*, and *E. quadrilineata,* in low frequencies. The data indicate that *F. vulgare* seeds from Uttar Pradesh have a high fungal burden and a considerable species diversity. The most prevalent and ecologically dominant contaminants were *A. niger*, *A. flavus*, and *Mucor* sp. (Table 4).

The six fennel seed samples subjected to mycological testing from Rajasthan revealed a high incidence, with total infection rates spanning from 90 to 100%. *A. niger* was the most abundant fungal species in all samples accounting to 66% in FOEV56, suggesting that the fungus is highly adaptable to diverse ecological habitats and climatic conditions. *A. flavus* (9–21%), *Penicillium* sp. (2.22–13%), *A. terreus*, *A. nidulans*, and *M. sterilia* were consistently present in moderate proportions across the samples. The mycological diversity of the seed samples was emphatic due to the identification of fungal taxa namely *S. cerevisiae*, *Mucor* sp., *A. fumigatus*, *Curvularia* sp., *Drechslera* sp., *A. awamori*, *A. tamarii*, and *E. quadrilineata*, nevertheless in lower frequencies. It is important to note that sample FOEV54 exhibited the highest species diversity (Table 5), dominated by *A. niger*.

In all, *Aspergillus* was the dominant genus, consistent with reports from other studies on stored spices (Hammami et al., 2014; Jeswal and Kumar, 2015; Mahata et al., 2022a). This emphasizes the urgent need for improved storage, moisture control, and better seed handling education for vendors and consumers. In a nutshell, this investigation highlights the necessity of standardized postharvest handling and storage protocols and illustrates the extensive variety of fungal contaminants linked to fennel seeds. These findings are a critical step toward assessing the toxigenic potential of the isolated fungi and their implications for public health and agriculture. Routine microbial screening and regulatory oversight are essential to ensure the safety and efficacy of fennel seeds in pharmaceutical and food sectors (Tosun and Arslan, 2013; Rawat et al., 2014).

The significance of environmental conditions, particularly temperature and humidity in the context of fungal contamination is underscored in this investigation. Nevertheless, it also highlights the significant influence of the supply chain, storage practices, and post-harvest management. Contamination in non-cultivating states suggests that the seeds are susceptible to fungal colonization beyond the harvesting stage, which raises concerns regarding quality control during transportation and retail storage. This necessitates the implementation of rigorous seed hygiene surveillance, particularly for products that are of medicinal and dietary importance, such as fennel.

In summary, this innovative study emphasizes the significance of monitoring fungal contamination in fennel seeds, particularly in non-producing regions where long-term storage and unfavorable environmental conditions may substantially contribute to contamination (Ahene et al., 2011). Further research should concentrate on the identification of the specific fungal genera that are involved, the assessment of mycotoxin occurrence, development of standard storage practices to reduce fungal proliferation and ensure the safety and efficacy of medicinal seeds (Zaied et al., 2010).

## Conclusion

As researchers delve into the fungal contamination studies of grains, and cereals in general and *F. vulgare* in particular, such mycological studies become more relevant, as they provide critical insights into the diversity, prevalence, and potential implications of seed-borne fungal pathogens in a crop that is both economically and medicinally valuable. Fennel’s post-harvest microbial quality has received comparatively little attention, despite its widespread use as a culinary spice and traditional remedy. The first exhaustive account of fungal contaminants affecting fennel seeds across various climatic regions and agricultural conditions in India has been made available in this study, which is a first report. Scientific data of 25 fungal species from 11 distinct genera, and their frequency of occurrence in *F. vulgare* could serve as an important document for researchers pursuing mycological and diversity studies. *A. niger* was the most prevalent contaminant, followed by *M. sterilia* and *A. flavus*. It is of particular concern that the latter is capable of producing aflatoxins, which are known to pose substantial health risks when consumed because of the carcinogenic, toxigenic and teratogenic potential (Mathew et al., 2026). Other notable species that have been identified include *Mucor* sp., *Penicillium* sp., *S. cerevisiae*, *F. oxysporum*, *A. alternata*, and *Drechslera* sp. These species contribute to the fungal burden and present variable levels of phytopathogenic and toxicological threats. An effective and comparative analysis of fungal profiles was facilitated by the fungal diversity of the collected samples, which ranged from fennel-growing states such as Rajasthan, Uttar Pradesh, and West Bengal to fennel non-growing states like Pondicherry and New Delhi.

It is intriguing that fungal contamination was prevalent in regions without fennel cultivation, indicating that environmental factors, storage conditions, and post-harvest management are critical factors in microbial proliferation. Variations in fungal burden and diversity across the states were associated with climatic variables, including temperature and relative humidity. For instance, in West Bengal, the prevalence of *M. sterilia* and other moisture-loving fungi was correlated with elevated humidity levels. Seeds which apparently appear healthy may pose toxic effects and serious health concerns and to human and animals. This further implies that healthy-looking seeds cannot be guaranteed and cleared for consumption, solely through traditional sorting methods. Rather, it is recommended that spice seed quality assurance protocols incorporate routine microbiological screening as a standard practice. The implications of these scientific findings are numerous. Fungal contamination can compromise seed germination rates, reduce crop vigor, and contribute to disease outbreaks in the field from an agricultural perspective. Other threats include, diminished marketability and value, from a commercial standpoint. Additionally, the presence of fungal spores may lead to trade rejections as a result of food safety regulations that pertain to mycotoxins. The presence of mycotoxigenic fungi, such as *A. flavus* and *F. oxysporum*, poses significant risks to consumers, particularly when fennel seeds are consumed raw or as part of traditional medicinal preparations.

Finally, the investigation underscores the detailed fungal ecosystem that is connected to *F. vulgare* seeds, which includes both saprophytic and potentially pathogenic fungi. The potential environmental, storage, or agronomic influences are also reflected in the dominance of specific species in the Indian states/ geographic regions. In order to prevent the dissemination of potentially post-harvest and storage fungi, adequate scientific practices need to be adopted and followed in every country. These findings also emphasize the necessity of rigorous seed health assessments through standard microbiological methods. Numerous small-scale producers and local vendors are generally unaware of the dangers associated with fungal contamination or the most effective methods for reducing it. Therefore, it is necessary to raise awareness and educate producers, seed traders, and consumers. Ultimately, this knowledge divide can be bridged through outreach programs and training workshops, which can contribute to more sustainable and safer spice production chains.

## Data Availability

The original contributions presented in the study are included in the article/supplementary material, further inquiries can be directed to the corresponding author/s.
